# *Pneumocystis pneumonia* during Postoperative Adjuvant Chemotherapy for Breast Cancer

**DOI:** 10.1155/2013/954346

**Published:** 2013-05-16

**Authors:** Tsuyoshi Shinohara, Makito Yasui, Hiroyuki Yamada, Yoshiro Fujimori, Kiyofumi Yamagishi

**Affiliations:** ^1^Department of Surgery, Hokushin General Hospital, 63-5-1 Nishi, Nakano-city, Nagano 383-8505, Japan; ^2^Department of Respiratory Medicine, Hokushin General Hospital, 63-5-1 Nishi, Nakano-city, Nagano 383-8505, Japan

## Abstract

A 72-year-old woman underwent a mastectomy with one-stage breast reconstruction using silicone implant for right breast cancer. Postoperatively, she had received adjuvant chemotherapy with fluorouracil, epirubicin, and cyclophosphamide (FEC regimen). She was admitted for febrile neutropenia after the third course of chemotherapy. She remained febrile for a week, and she complained of dyspnea on hospital day 8. Computed tomography scan demonstrated widespread patchy ground glass changes in both lungs and serum (1→3)-*β*-D-glucan was elevated to 20 pg/mL. Oral trimethoprim-sulfamethoxazole was started on the strong clinical suspicion of PCP, and the patient subsequently made a rapid recovery from fever and dyspnea.

## 1. Introduction

The efficacy of cyclophosphamide, methotrexate, and fluorouracil (CMF regimen) as an adjuvant chemotherapy for breast cancer was first reported by Bonadonna et al. in 1976 [1]. Since then, several trials have demonstrated that adjuvant systemic combination chemotherapy can reduce the risk of recurrence and prolong overall survival among patients with breast cancer [2–5].

Anthracyclines are an important component of adjuvant polychemotherapy, and fluorouracil, epirubicin, and cyclophosphamide (FEC regimen) have been established as one of the standard regimens for postoperative breast cancer patients. 

While the most common adverse effects in patients treated by FEC are nausea, leukocytopenia, and alopecia, opportunistic infections are not thought to be common in this population [6–8].

We describe here the case of a patient with severe *Pneumocystis pneumonia* (formerly *Pneumocystis carinii* pneumonia) (PCP) during postoperative adjuvant chemotherapy with FEC. 

## 2. Case Presentation

A 72-year-old postmenopausal woman visited our hospital for further evaluation to the following mammography at a medical checkup. The mammography had revealed a microlobulated tumor accompanied by fine linear branching calcifications showing a segmental distribution in the right breast.

No mass was palpable in the right breast, but core needle biopsy of the tumor under ultrasonography (US) showed malignant cells, which proved negative for hormonal receptors. Primary breast cancer was diagnosed based on the radiological and pathological findings. Total mastectomy with sentinel lymph node biopsy and a one-stage immediate breast reconstruction with a permanent silicone implant were performed. 

**Figure 1:**
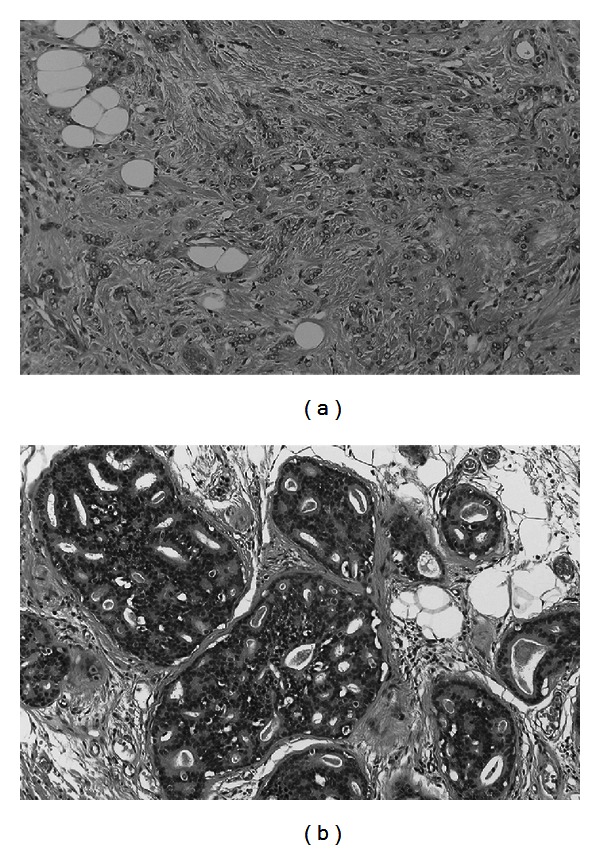
Microscopic findings of surgical specimens. (a) Invasive ductal carcinoma. (b) Intraductal spread.

Pathological examination of the resected specimens revealed invasive ductal carcinoma (scirrhous carcinoma) with intraductal spread measuring 6.0 × 5.0 cm, which was negative for human epidermal growth factor receptor type 2 (HER 2) (Figure 1). Sentinel lymph node biopsy yielded negative results. 

**Figure 2:**
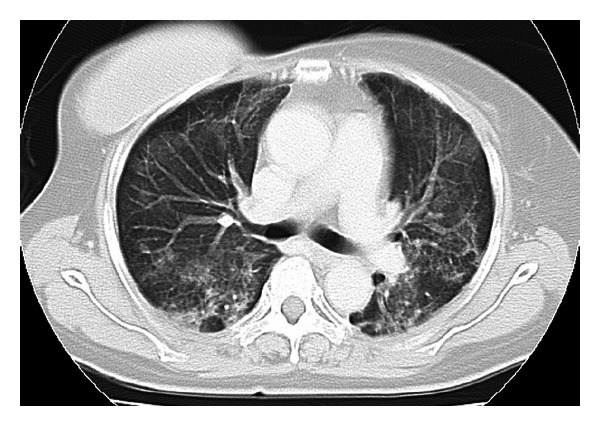
Computed tomography at the time when our patient complained of severe dyspnea. CT of the chest revealing widespread patchy ground glass changes in bilateral lobes.

**Figure 3:**
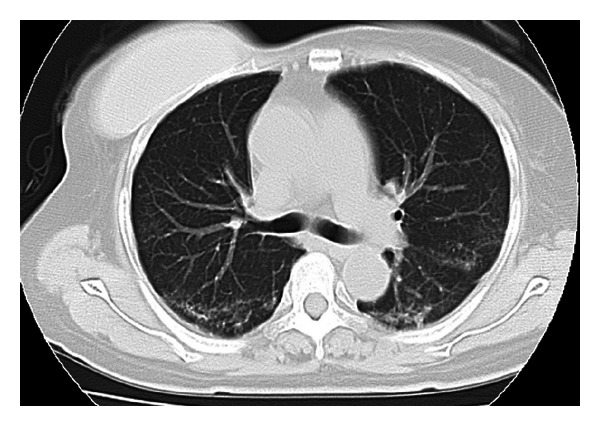
Computed tomography after the treatment for *pneumocystis pneumonia*. CT showing that pulmonary infiltration has almost disappeared as of 8 days after initiation of therapy.

**Figure 4:**
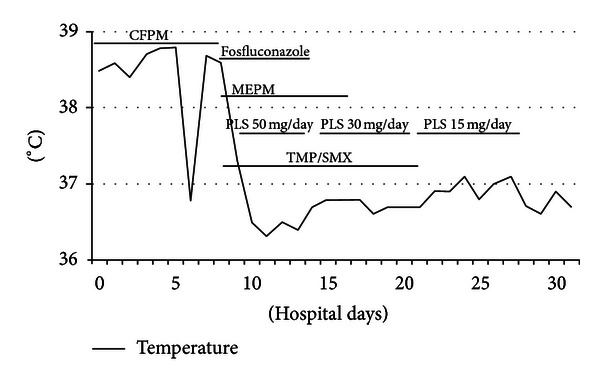
Clinical course. CFPM: cefepime dihydrochloride, MEPM: meropenem hydrate, PLS: prednisolone, and TMP/SMX: trimethoprim-sulfamethoxazole.

The patient was scheduled to receive six cycles of adjuvant chemotherapy, with each cycle comprising 5-Fluorouracil at 500 mg/m^2^, Epirubicin at 100 mg/m^2^, and Cyclophosphamide at 500 mg/m^2^ on day 1 within a 3-week period. The patient received 9.9 mg of intravenous dexamethasone on the day of chemotherapy and oral dexamethasone at 4 mg twice daily for 3 days after chemotherapy administration. The patient presented to our hospital due to fever and general fatigue 14 days after completing the third course of chemotherapy with FEC. Laboratory studies revealed a white blood cell count of 700/mm^3^, with 59% neutrophils. The patient was admitted for febrile neutropenia and intravenous cefepime dihydrochloride was commenced at 2.0 g two times daily. Granulocyte colony stimulating factor (G-CSF) therapy was also administered on hospital day 0 (Nartograstim 50 *μ*g subcutaneous injection). On hospital day 1, neutrophil count had recovered and G-CSF therapy was stopped. She remained febrile despite empiric antibiotic therapy for a week but showed no symptoms other than fever. On hospital day 8, the patient complained of severe dyspnea. Oxygen saturation was found to be 75% at rest on room air, rising to >90% with 100% O_2_ by nasal cannula. However, she showed no respiratory symptoms, including cough. Computed tomography (CT) demonstrated widespread patchy ground glass changes in both lungs (Figure 2). White blood cells and absolute neutrophil count were 7300/*μ*L and 6490/*μ*L, respectively, and serum (1→3)-*β*-D-glucan level was elevated to 20 pg/mL (normal, 0–11 pg/mL). Microscopic sputum examination was uninformative and blood cultures for bacteria and fungi yielded negative results. Viral serological testing for cytomegalovirus antigen pp65 was likewise negative. Examination of bronchoalveolar lavage fluid (BALF) was not performed because of severe respiratory failure. Serological testing for human immunodeficiency virus (HIV) was negative. After collecting sputum for polymerase chain reaction (PCR), she was treated with oral trimethoprim-sulfamethoxazole (TMP/SMX) in consideration of the possibility of PCP. Intravenous meropenem hydrate, fosfluconazole, and oral prednisolone were administered simultaneously. Although we obtained negative results for *Pneumocystis jiroveci* from sputum by PCR, the diagnosis of PCP was made based on the clinical course. Her temperature rapidly dropped to normal and dyspnea disappeared 4 days after starting TMP/SMX therapy. Chest CT after 8 days of TMP/SMX showed considerable improvement of pulmonary infiltrations (Figure 3). TMP/SMX therapy discontinued after 16 days. She was discharged without symptoms 32 days after the admission (Figure 4).

## 3. Discussion

PCP is an opportunistic infection caused by *Pneumocystis* jirovecii, a fungal organism with tropism for lung parenchyma [9]. PCP is predominantly seen in patients with acquired immunodeficiency syndrome (AIDS) but can also occur in patients with cancer. However, among patients with cancer, this disease is particularly common with hematological malignancies such as acute leukemia or malignant lymphoma and is less common in patients with solid tumors [10, 11]. Cases of PCP in patients with breast cancer are rare and most previously described patients with breast cancer who developed PCP were patients with high-dose chemotherapy for metastases [12–16]. Only two previous reports have described PCP occurring in breast cancer patients receiving postoperative adjuvant chemotherapy [17, 18].

PCP is well known as a major cause of mortality and morbidity in immunocompromised individuals. Outcomes appear worse in HIV-negative patients than in HIV-positive ones [7, 16, 18, 19], with a morbidity rate of 30%–60% in HIV-negative patients [9]. Early diagnosis and therapy are therefore vital. However, the diagnosis of PCP is difficult because microscopic examination is required to identify *P. jiroveci* from collected specimens such as induced sputum, BALF or lung tissue, since *P. jiroveci* cannot be cultured. The sensitivity of induced sputum is low, reported as about 50% [20]. Although examination of BALF offers good sensitivity, bronchoalveolar lavage is an invasive procedure with its own associated morbidity, particularly for patients with severe respiratory failure. Measurement of serum (1→3)-*β*-D-glucan levels is a noninvasive and useful tool for the diagnosis of PCP [21, 22], but (1→3)-*β*-D-glucan is a component of the cell wall in not only *P. jiroveci* but also various other fungi and thus reportedly offers only 76%–96% specificity [21]. Clinicians must therefore sometimes decide whether to treat PCP empirically on the basis of characteristic clinical and radiological presentations. 

Corticosteroids are thought to be one of the strongest predisposing factors for the development of PCP in patients who are not infected with HIV. In retrospective series, a daily corticosteroid dose equivalent to 16–30 mg of prednisone for a period of 8–12 weeks was associated with a significant risk of PCP in patients without AIDS [23]. Prophylactic administration of dexamethasone for antiemetic effect is recommended and well accepted, and the present patient had received only intermittent dexamethasone given for 4 days at the beginning of each cycle of chemotherapy. Although dexamethasone has approximately 7.5 times anti-inflammatory effect of prednisone, it is unknown whether short-duration, intermittent dosing with dexamethasone for antiemesis represents a risk factor for the development of opportunistic infections. 

PCP occurs most frequently when the T-helper cell count (CD4+) is <200 cells/mm^3^ [24, 25]. Lymphocyte depletion in patients receiving chemotherapy may correlate with the frequency and intensity of the dose [13], but clinicians tend to pay attention only to neutropenia, as represented by febrile neutropenia. Lymphocyte depletion may be induced more frequently or severely during dose-dense chemotherapy [26]. Although G-CSF therapy may immediately result in adequate recover from neutropenia, the degree of lymphocyte depletion may remain unaltered [26]. 

In conclusion, clinicians must always keep in mind that life-threatening PCP can be induced by dose-dense chemotherapy, even in the form of adjuvant chemotherapy in postoperative patients without cancer.
